# Dataset on the performance of a three phase induction motor under balanced and unbalanced supply voltage conditions

**DOI:** 10.1016/j.dib.2019.103947

**Published:** 2019-04-23

**Authors:** Aderibigbe Israel Adekitan, Isaac Samuel, Elizabeth Amuta

**Affiliations:** Department of Electrical and Information Engineering, Covenant University, Ota, Nigeria

**Keywords:** Motor performance characteristics, Power quality, Three phase induction motor, Positive and negative sequence component, Voltage unbalance

## Abstract

Three phase induction motors (TPIM) are extensively used for various applications in the industry for driving cranes, hoists, lifts, rolling mills, cooling fans, textile operations, and so forth. TPIM are designed to operate on balanced three phase power supply, but sometimes three phase supply line voltages to which the TPIM is connected may be unbalanced. In this data article, the operational data of a TPIM operating under changing voltage scenarios is profiled to determine the variations in the magnitude of the operational parameters of the motor. The magnitude of each of the line voltages was separately varied from the balanced state (0% unbalance) until 5% voltage unbalance condition was achieved, in line with the recommendations and guidelines of the National Electrical Manufactures Association. The motor parameters; both mechanical and electrical, at various slip values were collected in six sets for the 0%, 1%, 2%, 3%, 4%, and 5% unbalance voltage conditions. Frequency distributions and statistical analysis were carried out to identify the data pattern and data variation trends among the parameters in the dataset.

**Table:** Specifications table

Subject area	*Electrical Engineering*
More specific subject area	*Machines, Power Quality Analysis*
Type of data	*Figures, tables and spread sheet file*
How data was acquired	*The motor parameter data was acquired from the simulated operation of ATLAS Y225 M three phase induction motor under balanced and 1–5% unbalanced three phase supply conditions*
Data format	*Raw, analysed*
Experimental factors	*The data collected comprises the mechanical (positive and negative sequence torque, electromechanical power) and the electrical (rotor and stator current, winding copper losses, air gap power, real and reactive input power) motor parameters at various slip values, as the motor supply voltage unbalance increased from 0% to 5% unbalanced voltage.*
Experimental features	*Linear regression models, Frequency distributions, and Anova analysis were carried out to demonstrate data trends, and to identify the relationship among the motor data parameters*
Data source location	*Operational motor simulations at Covenant University, Nigeria*
Data accessibility	*The dataset is attached to this article in a spreadsheet file*
Related research article	*A. I. Adekitan, B. Adetokun, T. Shomefun, and A. Aligbe, “Cost implication of Line Voltage variation on Three Phase Induction Motor operation” TELKOMNIKA (Telecommunication Computing Electronics and Control), vol. 16, 2018.*

**Table:** 

**Value of the data** • Detailed TPIM operational parameters under changing voltage unbalance conditions are presented in this dataset. This data can be used for academic studies on voltage quality issues [1], [2], [3], [4], [5], and for demonstrating the concept of voltage unbalance in machine classes. • The tables, figures and frequency distribution presented, gives relevant information on the influence of voltage unbalance on motor parameters, and the undesirable effects of negative sequence motor components that results from unbalance supply. • The data and statistical analysis in this data article can be further developed to evolve a statistical model, data mining model [6] or an algorithm that can determine the voltage unbalance condition of a running TPIM based on monitored and profiled real time operational parameters of the motor. The statistical presentations in this article were evolved using similar methods to those found in [7]. • This data creates an opportunity for various statistical analyses to be performed for an improved understanding of voltage unbalance, and for discerning data patterns that can help in broadening available knowledge on the effects of unbalance voltage supply. • The availability of this data will trigger similar motor simulation, data collection and analysis, and this may provide a platform for extensive research collaboration.

## 1 Data

**Fig. 1:**
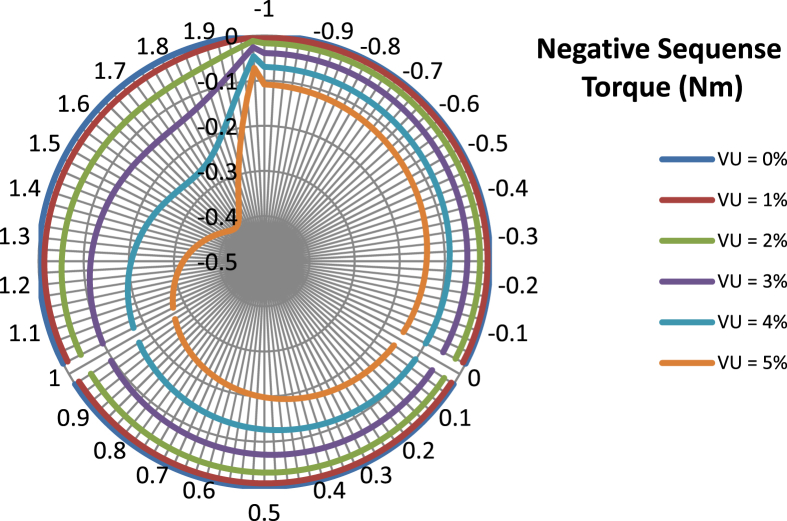
A radar plot of the Negative Sequence Torque with varying slip and unbalance.

**Fig. 2:**
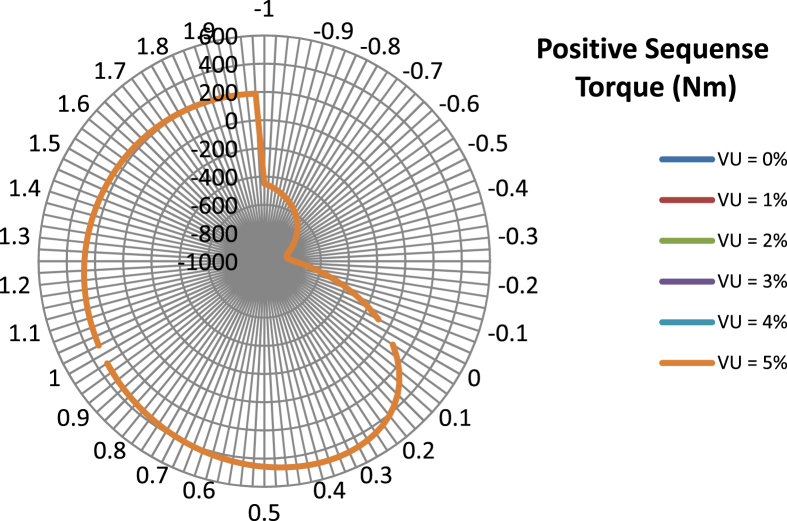
A radar plot of the Positive Sequence Torque with varying slip and unbalance.

**Fig. 3:**
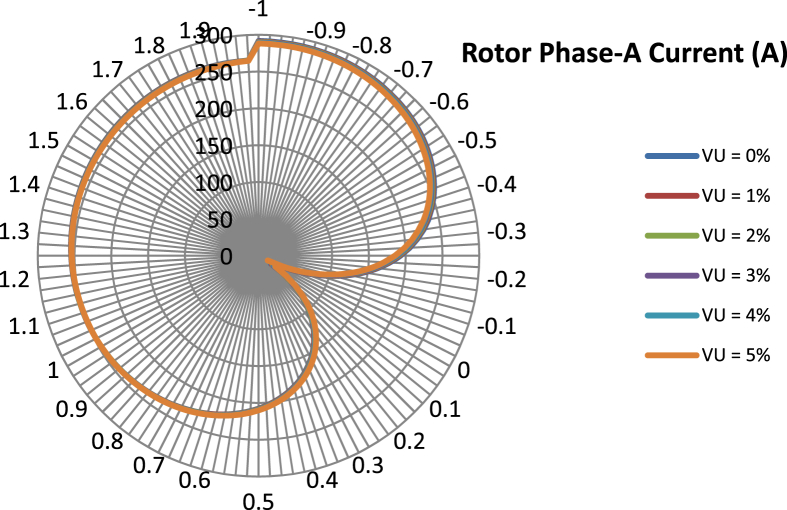
A radar plot of the Phase-A Rotor Current with varying slip and unbalance.

**Fig. 4:**
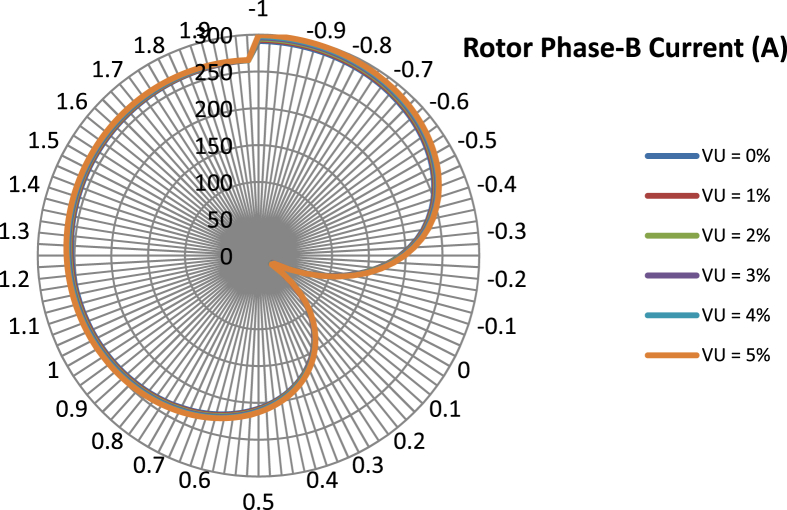
A radar plot of the Phase-B Rotor Current with varying slip and unbalance.

**Fig. 5:**
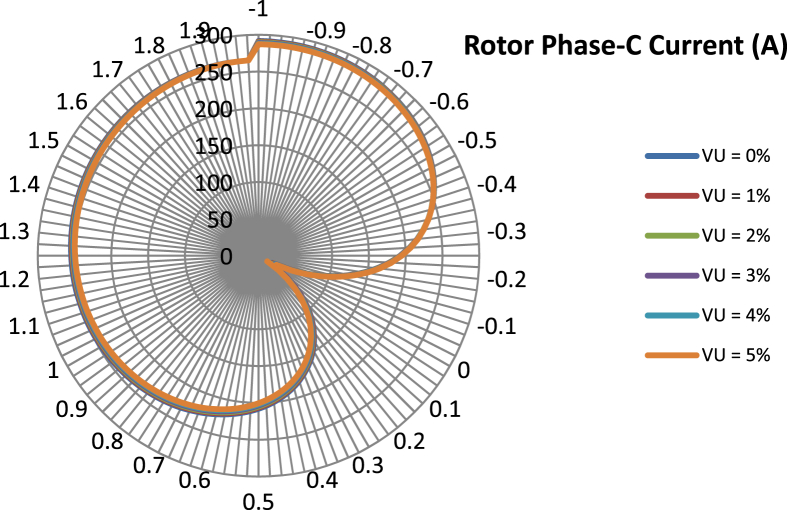
A radar plot of the Phase-C Rotor Current with varying slip and unbalance.

**Fig. 6:**
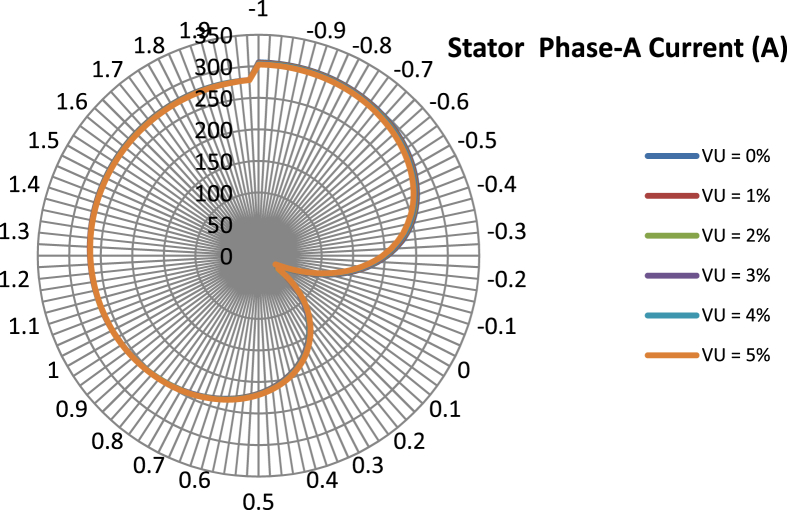
A radar plot of the Phase-A Stator Current with varying slip and unbalance.

**Fig. 7:**
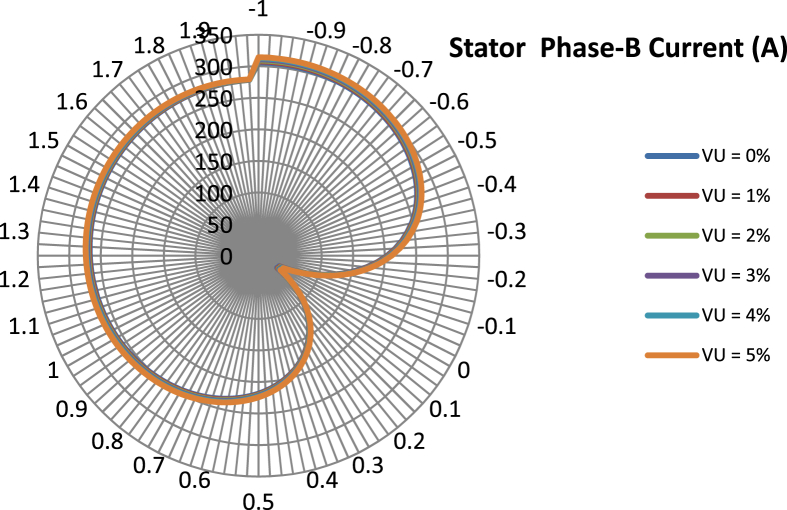
A radar plot of the Phase-B Stator Current with varying slip and unbalance.

**Fig. 8:**
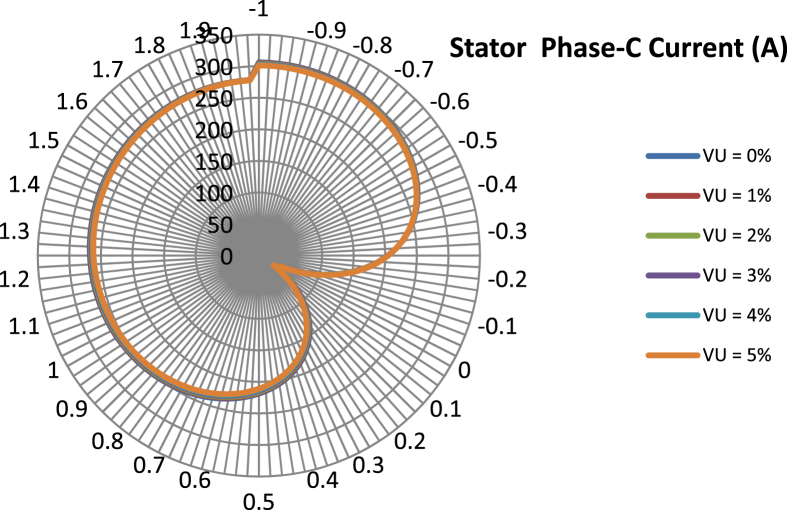
A radar plot of the Phase-C Stator Current with varying slip and unbalance.

**Fig. 9:**
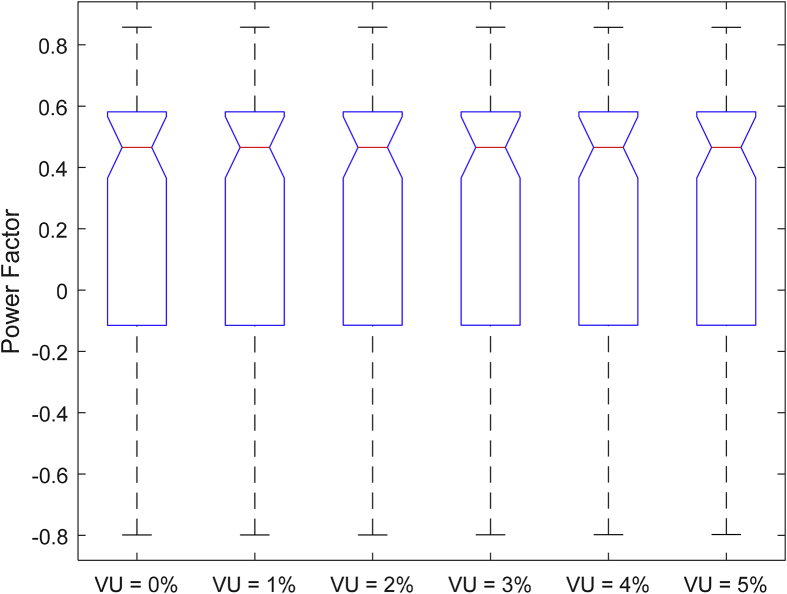
Boxplot of the Motor's Power Factor data set.

**Fig. 10:**
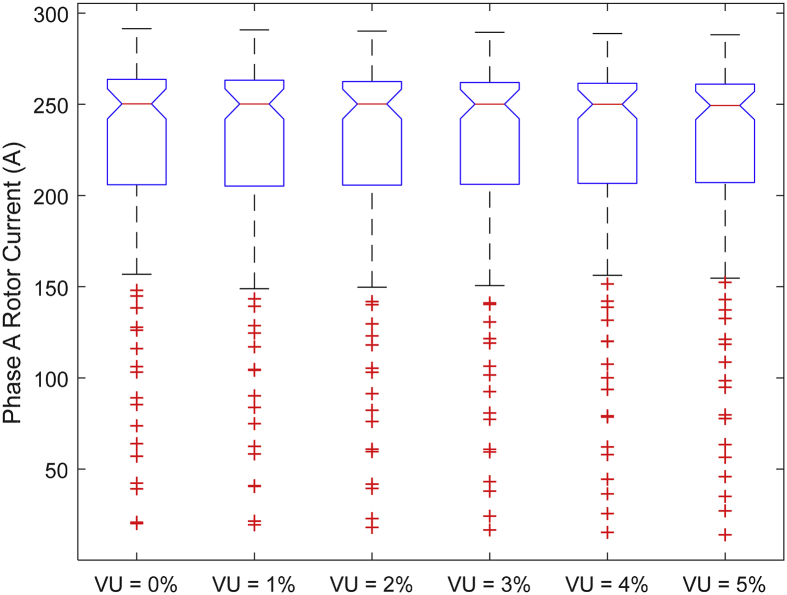
Boxplot of the Motor's Phase-A Rotor Current data set.

**Fig. 11:**
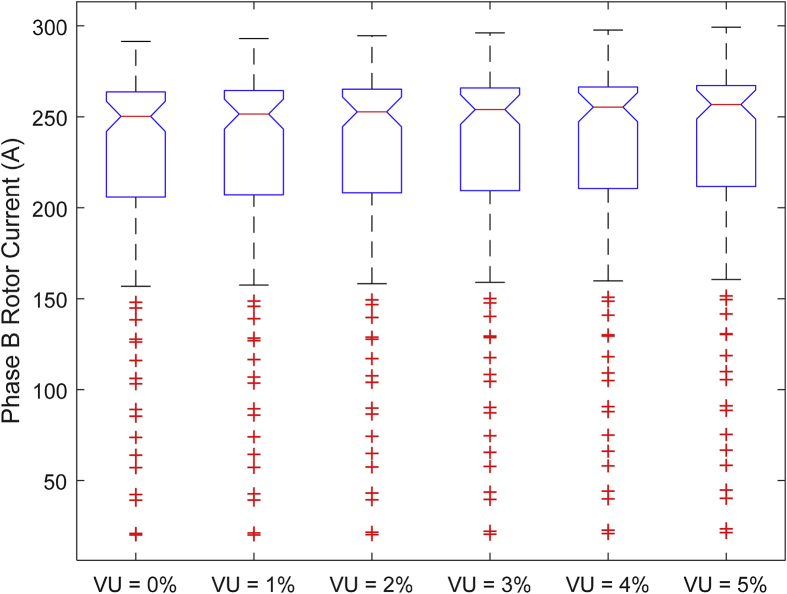
Boxplot of the Motor's Phase-B Rotor Current data set.

**Fig. 12:**
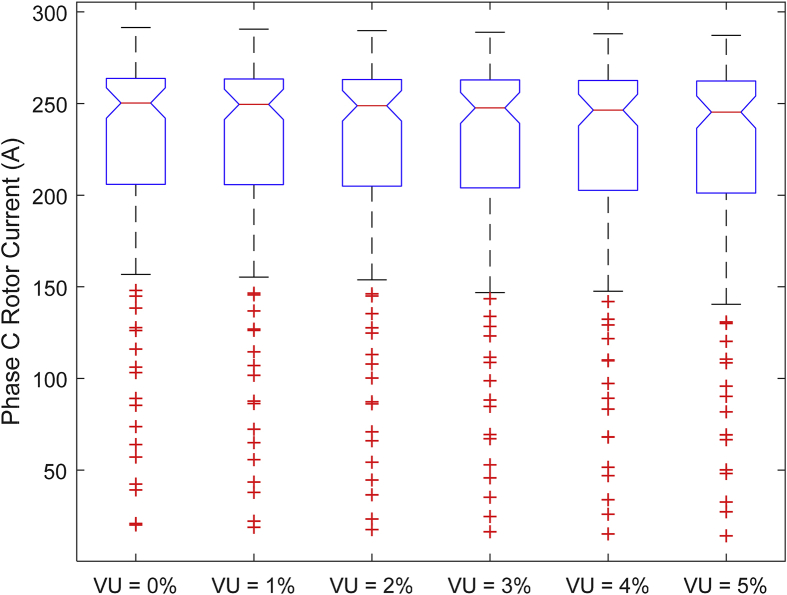
Boxplot of the Motor's Phase-C Rotor Current data set.

**Fig. 13:**
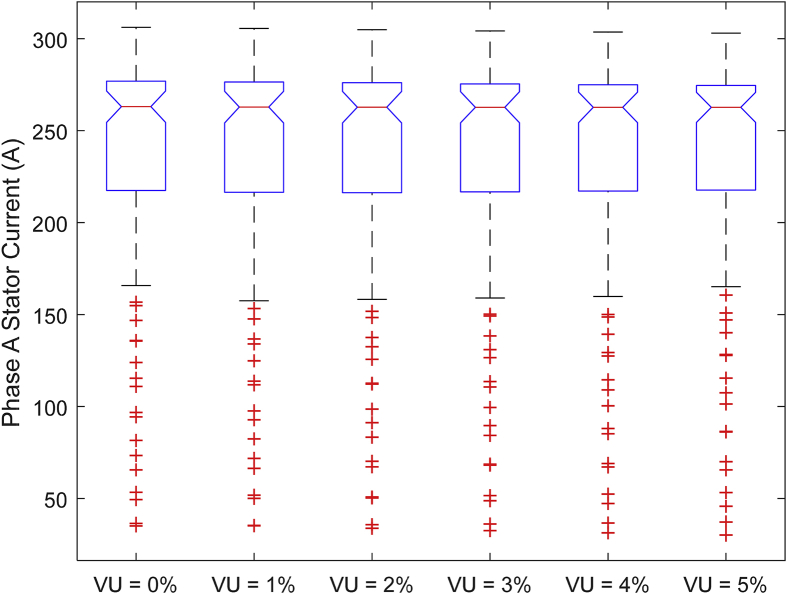
Boxplot of the Motor's Phase-A Stator Current data set.

**Fig. 14:**
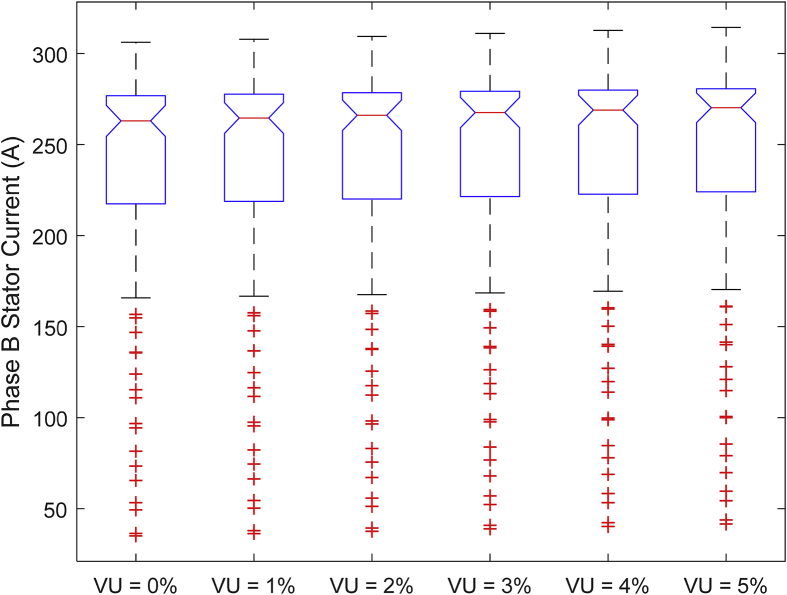
Boxplot of the Motor's Phase-B Stator Current data set.

**Fig. 15:**
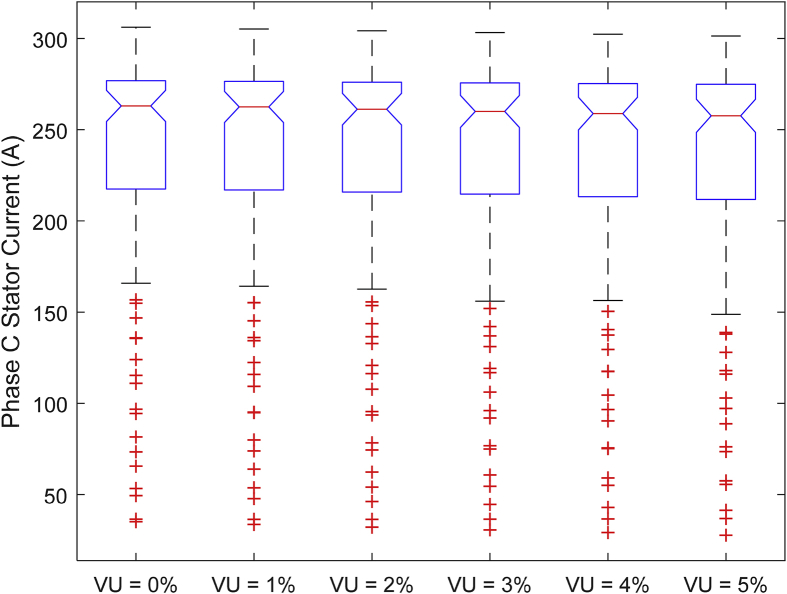
Boxplot of the Motor's Phase-C Stator Current data set.

**Fig. 16:**
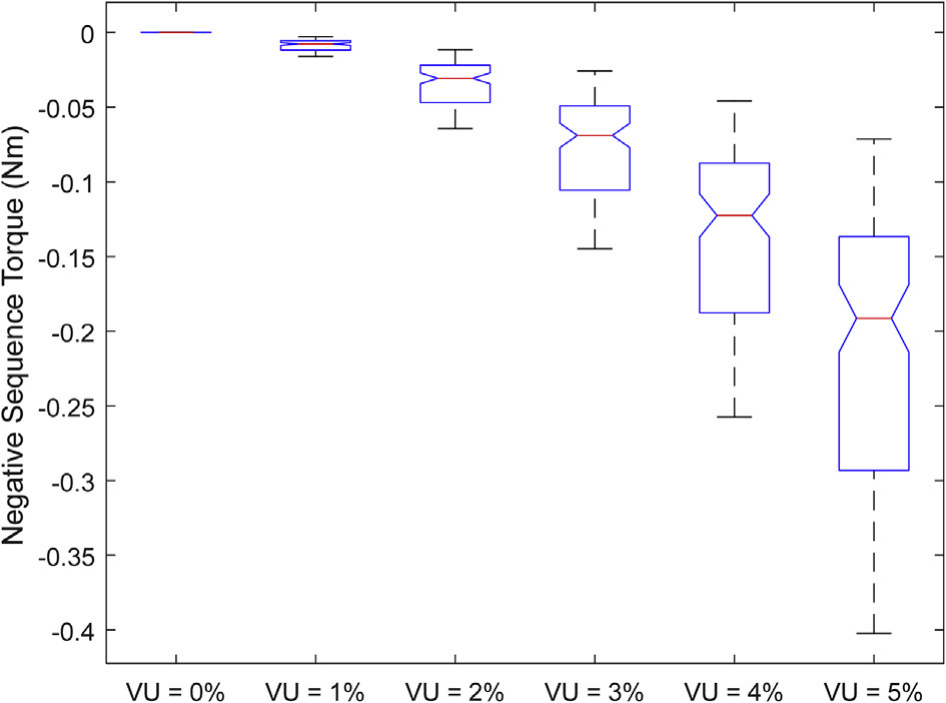
Boxplot of the Negative Sequence Torque data set.

**Fig. 17:**
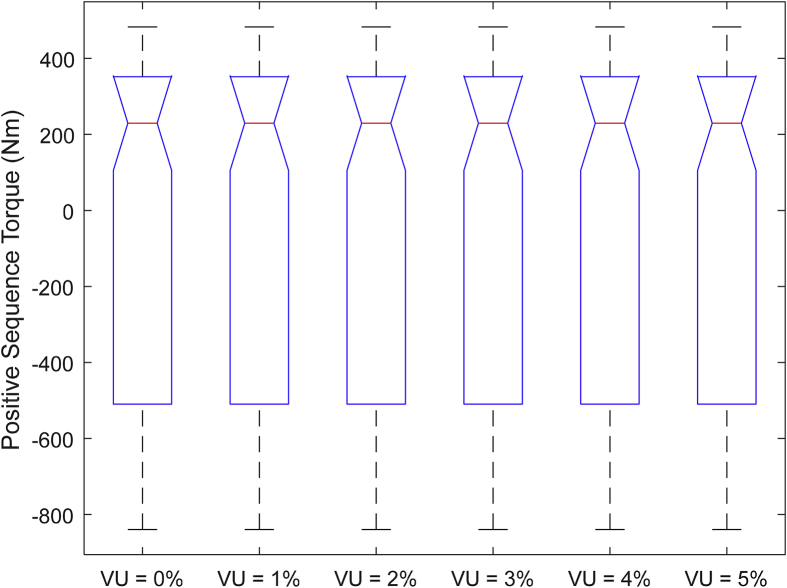
Boxplot of the Positive Sequence Torque data set.

**Fig. 18:**
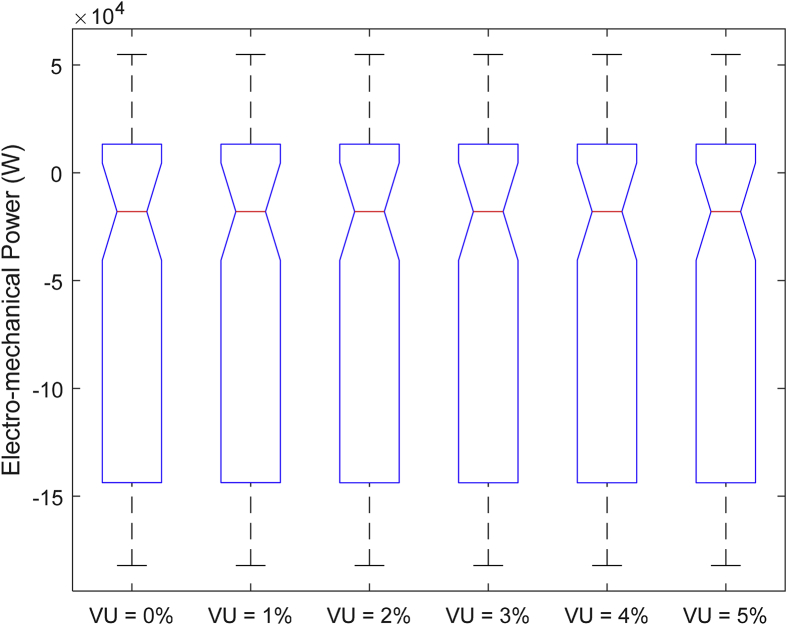
Boxplot of the Electromechanical Power data set.

**Fig. 19:**
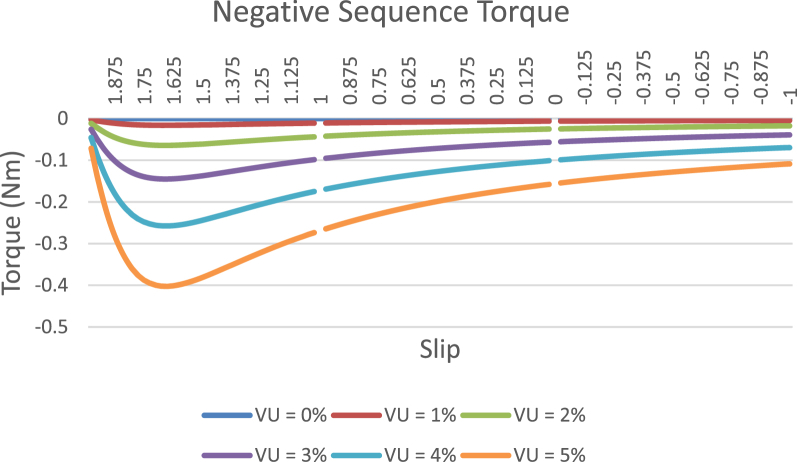
A plot of the Negative Sequence Torque with varying slip and unbalance.

**Fig. 20:**
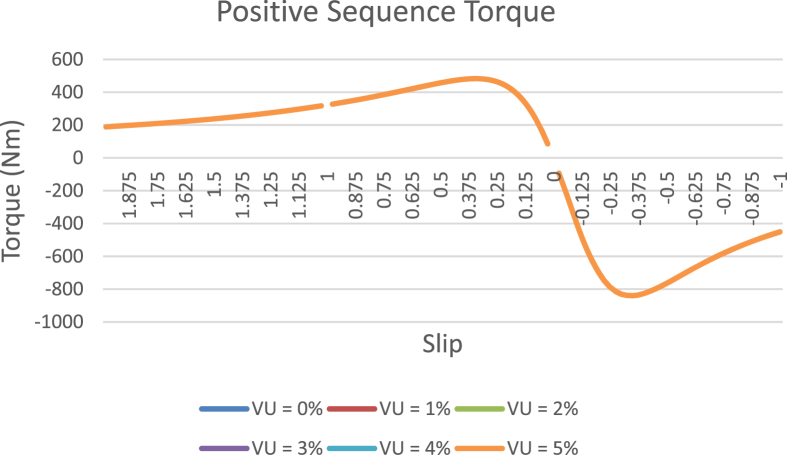
A plot of the Positive Sequence Torque with varying slip and unbalance.

The data presented in this article contains the key operational parameters of a TPIM as the supply voltage is varied from the balanced state to unbalance conditions (0%–5% unbalance) with reference to the National Electrical Manufacturers Association (NEMA) definition of voltage unbalance [8]. Table 1, Table 2, Table 3, Table 4, Table 5, Table 6 present the descriptive statistics of the rotor winding copper losses, the stator winding copper losses, the total energy losses in the motor, the real input power to the motor, the reactive input power, and the apparent power supplied to the motor. Fig. 1, Fig. 2, Fig. 3, Fig. 4, Fig. 5, Fig. 6, Fig. 7, Fig. 8 display the radar plots of the negative and positive sequence torque [8], [9], [10], [11], [12], [13], the motor current for the three phases, and the stator current for the three phases. Fig. 9, Fig. 10, Fig. 11, Fig. 12, Fig. 13, Fig. 14, Fig. 15, Fig. 16, Fig. 17, Fig. 18 present the comparative box plot of the motor performance parameters; both electrical and mechanical, as the voltage unbalance was increased from 0% to 5%. The line plot of the Negative Sequence Torque and the Positive Sequence Torque are shown in Fig. 19 and Fig. 20 respectively. Table 7 and Table 8 show the Anova test result for the negative and positive sequence torque data groups. Table 9, Table 10, Table 11, Table 12, Table 13, Table 14 present a quadratic regression analysis for predicting the total motor losses using the Negative (x_1_) and Positive (x_2_) Sequence Torque.

**Table 1:** Descriptive statistics of the total copper losses in the three rotor windings.

	VU = 0%	VU = 1%	VU = 2%	VU = 3%	VU = 4%	VU = 5%
*Mean*	45587.815	45589.46	45594.38	45602.58	45614.07	45628.83
*Sum*	5424950	5425145	5425731	5426707	5428074	5429831
*Min*	336.57834	338.5353	344.4062	354.191	367.8898	385.5025
*Max*	70742.079	70744.13	70750.26	70760.49	70774.82	70793.23
*Range*	70405.501	70405.59	70405.86	70406.3	70406.93	70407.73
*Variance*	375047155	3.75E+08	3.75E+08	3.75E+08	3.75E+08	3.75E+08
*Standard Deviation*	19366.134	19365.98	19365.51	19364.72	19363.62	19362.21
*Median*	52152.487	52154.12	52159	52167.15	52178.55	52193.21
*Excess Kurtosis*	−0.108107	−0.10808	−0.108	−0.10788	−0.1077	−0.10747
*Skewness*	−0.923071	−0.92306	−0.92302	−0.92295	−0.92286	−0.92275
*Count*	119	119	119	119	119	119

**Table 2:** Descriptive statistics of the total copper losses in the three stator windings.

	VU = 0%	VU = 1%	VU = 2%	VU = 3%	VU = 4%	VU = 5%
*Mean*	*43844.04*	*43845.61*	*43850.33*	*43858.2*	*43869.22*	*43883.39*
*Sum*	*5217440*	*5217628*	*5218189*	*5219126*	*5220437*	*5222123*
*Min*	*890.9139*	*892.7888*	*898.4132*	*907.7872*	*920.9108*	*937.7841*
*Max*	*67827.66*	*67829.62*	*67835.5*	*67845.3*	*67859.02*	*67876.66*
*Range*	*66936.75*	*66936.83*	*66937.09*	*66937.51*	*66938.11*	*66938.87*
*Variance*	*3.39E* + *08*	*3.39E* + *08*	*3.39E* + *08*	*3.39E* + *08*	*3.39E* + *08*	*3.39E* + *08*
*Standard Deviation*	*18403.14*	*18402.99*	*18402.55*	*18401.81*	*18400.77*	*18399.45*
*Median*	*50054.23*	*50056.15*	*50061.91*	*50071.51*	*50084.94*	*50102.22*
*Excess Kurtosis*	*−0.11621*	*−0.11619*	*−0.11611*	*−0.11599*	*−0.11581*	*−0.11558*
*Skewness*	*−0.91468*	*−0.91466*	*−0.91462*	*−0.91455*	*−0.91446*	*−0.91434*
*Count*	*119*	*119*	*119*	*119*	*119*	*119*

**Table 3:** Descriptive statistics of the total energy loss in the motor.

	VU = 0%	VU = 1%	VU = 2%	VU = 3%	VU = 4%	VU = 5%
*Mean*	*89431.85*	*89435.07*	*89444.71*	*89460.78*	*89483.29*	*89512.22*
*Sum*	*10642390*	*10642773*	*10643920*	*10645833*	*10648511*	*10651954*
*Min*	*1227.492*	*1231.324*	*1242.819*	*1261.978*	*1288.801*	*1323.287*
*Max*	*138569.7*	*138573.7*	*138585.8*	*138605.8*	*138633.8*	*138669.9*
*Range*	*137342.2*	*137342.4*	*137342.9*	*137343.8*	*137345*	*137346.6*
*Variance*	*1.43E* + *09*	*1.43E* + *09*	*1.43E* + *09*	*1.43E* + *09*	*1.43E* + *09*	*1.43E* + *09*
*Standard Deviation*	*37769.08*	*37768.77*	*37767.86*	*37766.34*	*37764.2*	*37761.47*
*Median*	*102146.8*	*102150*	*102159.6*	*102175.6*	*102197.9*	*102226.6*
*Excess Kurtosis*	*−0.11205*	*−0.11203*	*−0.11195*	*−0.11183*	*−0.11165*	*−0.11142*
*Skewness*	*−0.91899*	*−0.91898*	*−0.91894*	*−0.91887*	*−0.91878*	*−0.91866*
*Count*	*119*	*119*	*119*	*119*	*119*	*119*

**Table 4:** Descriptive statistics of the real input power (W).

	VU = 0%	VU = 1%	VU = 2%	VU = 3%	VU = 4%	VU = 5%
*Mean*	*44460.16*	*44463.11*	*44471.97*	*44486.73*	*44507.39*	*44533.96*
*Sum*	*5290759*	*5291110*	*5292164*	*5293921*	*5296380*	*5299542*
*Min*	*−93570.9*	*−93568.1*	*−93559.8*	*−93545.8*	*−93526.4*	*−93501.3*
*Max*	*106385*	*106388*	*106397.2*	*106412.5*	*106433.8*	*106461.3*
*Range*	*199955.9*	*199956.2*	*199957*	*199958.3*	*199960.2*	*199962.6*
*Variance*	*4.96E* + *09*	*4.96E* + *09*	*4.96E* + *09*	*4.96E* + *09*	*4.96E* + *09*	*4.96E* + *09*
*Standard Deviation*	*70413.4*	*70413.56*	*70414.04*	*70414.83*	*70415.94*	*70417.37*
*Median*	*88479.82*	*88482.97*	*88492.4*	*88508.12*	*88530.14*	*88558.44*
*Excess Kurtosis*	*−1.05034*	*−1.05035*	*−1.05036*	*−1.05038*	*−1.05041*	*−1.05044*
*Skewness*	*−0.80013*	*−0.80013*	*−0.80012*	*−0.80011*	*−0.8001*	*−0.80008*
*Count*	*119*	*119*	*119*	*119*	*119*	*119*

**Table 5:** Descriptive statistics of the reactive input power (VAR).

	VU = 0%	VU = 1%	VU = 2%	VU = 3%	VU = 4%	VU = 5%
*Mean*	*146464.6*	*146469.7*	*146485.1*	*146510.8*	*146546.8*	*146593*
*Sum*	*17429284*	*17429896*	*17431730*	*17434787*	*17439067*	*17444570*
*Min*	*20739.46*	*20745.5*	*20763.6*	*20793.77*	*20836.01*	*20890.32*
*Max*	*220055.4*	*220061.7*	*220080.6*	*220112.1*	*220156.1*	*220212.8*
*Range*	*199315.9*	*199316.2*	*199317*	*199318.3*	*199320.1*	*199322.5*
*Variance*	*2.99E* + *09*	*2.99E* + *09*	*2.99E* + *09*	*2.99E* + *09*	*2.99E* + *09*	*2.99E* + *09*
*Standard Deviation*	*54656.33*	*54655.94*	*54654.78*	*54652.84*	*54650.13*	*54646.64*
*Median*	*163776.8*	*163781.8*	*163796.7*	*163821.6*	*163856.5*	*163901.3*
*Excess Kurtosis*	*−0.20388*	*−0.20386*	*−0.20379*	*−0.20368*	*−0.20352*	*−0.20332*
*Skewness*	*−0.81939*	*−0.81937*	*−0.8193*	*−0.8192*	*−0.81905*	*−0.81886*
*Count*	*119*	*119*	*119*	*119*	*119*	*119*

**Table 6:** Descriptive statistics of the apparent input power (VA).

	VU = 0%	VU = 1%	VU = 2%	VU = 3%	VU = 4%	VU = 5%
*Mean*	*170413*	*170418*	*170433.2*	*170458.5*	*170494*	*170539.6*
*Sum*	*20279143*	*20279745*	*20281553*	*20284565*	*20288783*	*20294207*
*Min*	*25222.29*	*25228.88*	*25248.66*	*25281.63*	*25327.78*	*25387.12*
*Max*	*220074.7*	*220080.9*	*220099.7*	*220131*	*220174.9*	*220231.3*
*Range*	*194852.4*	*194852.1*	*194851.1*	*194849.4*	*194847.1*	*194844.1*
*Variance*	*2.29E* + *09*	*2.29E* + *09*	*2.29E* + *09*	*2.29E* + *09*	*2.29E* + *09*	*2.29E* + *09*
*Standard Deviation*	*47810.04*	*47810.28*	*47810.98*	*47812.16*	*47813.8*	*47815.9*
*Median*	*189054.5*	*189058.9*	*189072.3*	*189094.4*	*189125.5*	*189165.4*
*Excess Kurtosis*	*1.534721*	*1.534732*	*1.534763*	*1.534814*	*1.534885*	*1.534976*
*Skewness*	*−1.50958*	*−1.50959*	*−1.50961*	*−1.50964*	*−1.50969*	*−1.50974*
*Count*	*119*	*119*	*119*	*119*	*119*	*119*

**Table 7:** ANOVA – negative sequence torque (VU = 0–5%).

Source	Sum of Squares	Degree of Freedom	Mean Squares	F-Statistics	Prob > F
Groups	4.2974	5	0.85949	369.6736	6.83E-194
Error	1.6321	702	0.002325		
Total	5.9296	707

**Table 8:** ANOVA – Positive Sequence Torque (VU = 0–5%).

Source	Sum of Squares	Degree of Freedom	Mean Squares	F-Statistics	Prob > F
Groups	4.25E-25	5	8.49E-26	3.95E-31	1
Error	1.51E+08	702	215110.7		
Total	1.51E+08	707

**Table 9:** Regression - Total Loss prediction using Negative and Positive Sequence Torque (VU = 0%).

Estimated Coefficients				
(Intercept)	Estimate	SE	tStat	pValue
	1.02E+05	7101.5	14.306	4.92E-27
**x**_**1**_	0	0	–	–
**x**_**2**_	−39.087	12.088	−3.2336	0.0016064
**x**_**1**_**x**_**2**_	0	0	–	–
**x**_**1**_^**2**^	0	0	–	–
**x**_**2**_^**2**^	−0.057192	0.029287	−1.9528	0.053333

Number of observations (N): 118, Error degrees of freedom (EDF): 115.

Root Mean Squared (RMS) Error: 3.65e+04.

R-squared (R^2^): 0.0913, Adjusted R-Squared (Adj. R^2^): 0.0755.

F-statistic vs. constant model: 5.78, p-value = 0.00406.

**Table 10:** Regression - Total Loss prediction using Negative and Positive Sequence Torque (VU = 1%).

Estimated Coefficients				
(Intercept)	Estimate	SE	tStat	pValue
	1.71E+05	21407	7.9873	1.34E-12
x_1_	2.58E+07	4.89E+06	5.2751	6.54E-07
x_2_	−571.64	40.904	−13.975	2.66E-26
x_1_x_2_	−91951	6760.7	−13.601	1.82E-25
x_1_^2^	6.39E+08	2.24E+08	2.8462	0.0052635
x_2_^2^	−0.037906	0.018781	−2.0184	0.04594

N: 118, EDF: 112.

RMS Error: 2.03e+04.

R^2^: 0.725, Adj. R^2^: 0.712.

F-statistic vs. constant model: 59, p-value = 8.73e-30.

**Table 11:** Regression - Total Loss prediction using Negative and Positive Sequence Torque (VU = 2%).

Estimated Coefficients				
(Intercept)	Estimate	SE	tStat	pValue
	1.71E+05	21404	7.9885	1.33E-12
x_1_	6.45E+06	1.22E+06	5.2756	6.53E-07
x_2_	−571.66	40.9	−13.977	2.64E-26
x_1_x_2_	−22989	1690	−13.603	1.80E-25
x_1_	3.99E+07	1.40E+07	2.8462	0.0052635
x_2_^2^	−0.037902	0.018779	−2.0184	0.045944

N: 118, EDF: 112.

RMS Error: 2.03e+04.

R^2^: 0.725, Adj. R^2^: 0.712.

F-statistic vs. constant model: 59, p-value = 8.66e-30.

**Table 12:** Regression - Total Loss prediction using Negative and Positive Sequence Torque (VU = 3%).

Estimated Coefficients				
(Intercept)	Estimate	SE	tStat	pValue
	1.71E+05	21401	7.9905	1.31E-12
x_1_	2.86E+06	5.43E+05	5.2764	6.51E-07
x_2_	−571.69	40.893	−13.98	2.60E-26
x_1_x_2_	−10218	750.99	−13.606	1.77E-25
x_1_^2^	7.88E+06	2.77E+06	2.8462	0.0052635
x_2_^2^	−0.037896	0.018776	−2.0184	0.045944

N: 118, EDF: 112.

RMS Error: 2.03e+04.

R^2^: 0.725, Adj. R^2^: 0.712.

F-statistic vs. constant model: 59, p-value = 8.54e-30.

**Table 13:** Regression - Total Loss prediction using Negative and Positive Sequence Torque (VU = 4%).

Estimated Coefficients				
(Intercept)	Estimate	SE	tStat	pValue
	1.71E+05	21396	7.9934	1.29E-12
x_1_	1.61E+06	3.05E+05	5.2775	6.48E-07
x_2_	−571.73	40.884	−13.984	2.54E-26
x_1_x_2_	−5748	422.33	−13.61	1.74E-25
x_1_^2^	2.49E+06	8.76E+05	2.8462	0.0052635
x_2_^2^	−0.037887	0.018771	−2.0184	0.045944

N: 118, EDF: 112.

RMS Error: 2.03e+04.

R^2^: 0.725, Adj. R^2^: 0.713.

F-statistic vs. constant model: 59, p-value = 8.37e-30.

**Table 14:** Regression - Total Loss prediction using Negative and Positive Sequence Torque (VU = 5%).

Estimated Coefficients				
(Intercept)	Estimate	SE	tStat	pValue
	1.71E+05	21389	7.997	1.27E-12
x_1_	1.03E+06	1.95E+05	5.2789	6.44E-07
x_2_	−571.79	40.872	−13.99	2.47E-26
x_1_x_2_	−3679.1	270.21	−13.616	1.69E-25
x_1_^2^	1.02E+06	3.59E+05	2.8462	0.0052635
x_2_^2^	−0.037876	0.018766	−2.0184	0.045944

N: 118, EDF: 112.

RMS Error: 2.03e+04.

R^2^: 0.725, Adj. R^2^: 0.713.

F-statistic vs. constant model: 59.1, p-value = 8.16e-30.

## 2 Experimental design, materials and methods

The voltage unbalance scenarios were created by separately varying the line voltages from the rated value such that the three line voltages are no longer equal in magnitude [14], [15], [16]. The operational data was acquired from the simulated operation of a 415V TPIM with the following per unit specifications: Xm = 7.9626Ω, Xs = 0.3965Ω, Xr = 0.3965Ω, Rr = 0.2775Ω, Rs = 0.2412Ω. The voltage supply was varied from the balanced state (0% voltage unbalance) until it reached the NEMA recommended 5% maximum voltage unbalance level. A TPIM can operate in three modes depending on the values of the slip, and these modes are: generating mode (−1 <slip<0), motoring mode (0 < slip<1) and the plugging mode (1 < slip<2). The data presented in this data article spreads across a slip spectrum of −1 to 2, covering the three operational modes of a TPIM. The data captures both the electrical (rotor current, stator current, winding copper losses, real input power, reactive input power, the apparent power, and air gap power) and the mechanical (torque and electromechanical power) motor parameters. These set of parameters were collected and profiled for the six voltage supply scenarios (0%, 1%, 2%, 3%, 4%, and 5% unbalance voltage) and various frequency distributions and statistical analysis were performed to identify trends and data pattern. The data was processed using MATLAB to evolve the Anova for the negative and the positive sequence torques. The Anova test indicates the statistical variation of the torque data among the six groups (0%, 1%, 2%, 3%, 4%, and 5% unbalance voltage operation). Likewise, a quadratic regression analysis was performed to identify the correlation, if any, between the sequence torques and the motor losses.

Regression model (Quadratic).

(1) $$y=a+bx_{1}+cx_{2}+dx_{1}\cdot x_{2}+ex_{1}^{2}+fx_{2}^{2}$$

## References

[1] Gnacinski P., Tarasiuk T. (2016). Energy-efficient operation of induction motors and power quality standards. Electr. Power Syst. Res..

[2] Adekitan A.I., Adetokun B., Shomefun T., Aligbe A. (2018). Cost implication of line voltage variation on three phase induction motor operation. TELKOMNIKA (Telecommunication Computing Electronics and Control).

[3] Abdulkareem A., Awosope C.O.A., Adoghe A.U., Alayande S.A. (2016). Investigating the effect of asymmetrical faults at some selected buses on the performance of the Nigerian 330-kV transmission system. Int. J. Appl. Eng. Res..

[4] Samuel I.A., Katende J., Awosope C.O., Awelewa A.A. (2017). Prediction of voltage collapse in electrical power system networks using a new voltage stability index. Int. J. Appl. Eng. Res..

[5] Adekitan A.I. (2018). Supply instability induced torque variations of a three phase asynchronous motor. Int. J. Mech. Eng. Technol..

[6] Adekitan Aderibigbe Israel, Adewale Adeyinka, Olaitan Alashiri (2019). Determining the operational status of a Three Phase Induction Motor using a predictive data mining model. Int. J. Power Electron. Drive Syst..

[7] Adekitan A.I., Adetokun B.B., Aligbe A., Shomefun T., Orimogunje A. (2018/10/01/2018). Data based investigation of the energy metering type, billing and usage of sampled residents of Ota Community in Nigeria. Data in Brief.

[8] Pillay P., Manyage M. (2001). Definitions of voltage unbalance. IEEE Power Eng. Rev..

[9] Pillay P., Hofmann P., Manyage M. (2002). Derating of induction motors operating with a combination of unbalanced voltages and over or undervoltages. IEEE Trans. Energy Conservation.

[10] Siddique M., Yadava G.S., Sing B. (2004). Effects of voltage unbalance on induction motors. Conference Record of the 2004 IEEE International Symposium on Electrical Insulation.

[11] Annette V.J. (2000). Voltage Unbalance: Power Quality Issues, Related Standards and Mitigation Techniques.

[12] Williams J.E. (1954). Operation of 3-phase induction motors on unbalanced voltages [includes discussion]," Transactions of the American Institute of Electrical Engineers. Part III: Power Apparatus and Systs..

[13] Adekitan A., Ogunjuyigbe A.S.O., Ayodele T.R. (2019). The impact of supply phase shift on the three phase induction motor operation. Eng. Rev..

[14] Annette J.V., Basudeb B.B. (2001). Assessment of voltage unbalance. IEEE Trans. Power Deliv..

[15] Bossio G.R., Angelo C.H.D., Donolo P.D., Castellino A.M., Garcia G.O. (2009). Effects of voltage unbalance on IM power, torque and vibrations. 2009 IEEE International Symposium on Diagnostics for Electric Machines.

[16] Faiz J., Ebrahimpour H. (2005). Precise derating of three-phase induction motors with unbalanced voltages.

